# Prognostic factors in nasopharyngeal carcinoma with synchronous liver metastasis: a retrospective study for the management of treatment

**DOI:** 10.1186/1748-717X-8-272

**Published:** 2013-11-19

**Authors:** Yun-Ming Tian, Lei Zeng, Feng-Hua Wang, Shuai Liu, Ying Guan, Tai-Xiang Lu, Fei Han

**Affiliations:** 1 State Key Laboratory of Oncolo-gy in South China, Collaborative Innovation Center of Cancer Medicine, Sun Yat-sen University Cancer Center, Guangzhou, PR China; 2 Department of Radiation Oncology, Cancer Center of Sun Yat-Sen University, 651 Dong Feng, Road East, Guangzhou 510060, Guangdong Province, PR China

**Keywords:** Nasopharyngeal carcinoma, Liver metastasis, Prognosis, Response, Lactate dehydrogenase, Local therapy

## Abstract

**Purpose:**

To retrospectively analyze the prognosis of patients with nasopharyngeal carcinoma (NPC) initially presenting with liver metastasis, in order to identify independent prognostic factors to facilitate management of treatment.

**Methods:**

Eighty-five patients with untreated NPC and synchronous liver metastasis, initially diagnosed between January 2000 and December 2009, were selected for this retrospective study. Seventy-eight received systemic chemotherapy, 32 underwent subsequent radiotherapy of the primary tumor, and 18 received local therapy for metastatic lesions. Clinical features, laboratory parameters and treatment modalities were compared by univariate and multivariate analyses.

**Results:**

The median survival time was 19.0 months and the 3-year overall survival rate was 14.1%. The overall response and disease control rates were 70.4% and 86.4%, respectively. Significant predictors of short survival were KPS ≤ 70 (*P* = 0.03), serum lactate dehydrogenase levels >245 IU/l (*P* = 0.01) and poor response to chemotherapy (*P* < 0.01). In contrast, significantly longer survival rates were achieved by patients having at least six chemotherapy cycles compared to those receiving <6 cycles (3-year OS: 18.3% vs. 7.1%; *P* < 0.01), and patients receiving radiotherapy of the primary tumor following complete or partial response to chemotherapy (3-year OS: 30.8% vs. 3.8%, *P* < 0.01).

**Conclusions:**

Five key independent factors were identified and sub-classified as potential prognostic indicators for NPC with liver metastases. Progressive treatments of systemic chemotherapy and radiotherapy at the primary tumor could prolong survival in the subset of patients having fewer negative prognosticators.

## Introduction

Nasopharyngeal carcinoma (NPC) is a disease with a distinct racial and geographical distribution, with the highest incidence rate in Southeast Asia. NPC is biologically different from other squamous cell cancers of the head and neck, with approximately 95% classed as undifferentiated carcinomas (WHO stage III). It also has the highest propensity for lymphatic spread and distant metastases [1-4]. Over 70% of patients with NPC have neck masses of 6%-15% and present with synchronous distant metastasis at initial diagnosis, the most common sites being bone, lung and liver [5-7]. Several reports have indicated that liver metastasis may be an independent negative prognostic factor compared to bone or lung metastasis [8-12].

Studies relating to NPC with liver metastasis are limited, and few distinguish between patients with synchronous liver metastasis and those who develop liver metastasis following treatment. Patients presenting with NPC and liver metastasis at initial diagnosis undergo different treatment regimens, and have different survival rates, compared to those with subsequent liver metastasis [13]. Although palliative chemotherapy has been shown to achieve high objective response rates, recurrence frequently occurs after chemotherapy ceases. However, the application of local therapy of the primary tumor and metastatic liver lesions remains controversial [14-16].

The purpose of this study was to identify potential prognostic factors in NPC with synchronous liver metastasis by retrospectively analyzing patients’ clinical characteristics, treatment modalities and survival. The results could contribute to future management.

## Materials and methods

### Patients and selection criteria

Patients with NPC presenting with liver metastasis at initial diagnosis were referred to Sun Yat-Sen University Cancer Center between January 2000 and December 2009. The selection criteria were as follows: pathologically confirmed NPC in the nasopharynx; diagnosis of liver metastasis based on physical examination and imaging; adequate renal function as demonstrated by a creatinine clearance rate of ≥60 mL/min; complete follow-up and clinical data, including laboratory and imaging data. Patients with other malignancies or unstable cardiac disease requiring treatment were excluded.

### Baseline and treatment evaluations

Each patient received a pretreatment evaluation which included complete history, physical examination, hematology and biochemistry profiling including liver and renal functions, Epstein-Barr virus serology, chest radiographs, sonography and CT of the abdomen, whole-body bone scan and MRI of the nasopharynx and neck. Imaging of the abdomen was performed after every two courses of chemotherapy, and then every 3 months during follow-up. The median follow-up period was 17.0 months. Survival status was verified on December 31, 2012, by direct telecommunication with the patient or their family and by checking the clinic attendance records.

### Clinicopathological, laboratory and survival assessments

Overall survival (OS) was measured from diagnosis until the date of death from any cause. Patients were censored if remained alive at the time of the last follow-up. Objective response was measured according to the Response Evaluation Criteria in Solid Tumors (RECIST). Patients’ characteristics including Karnosky performance score (KPS), gender and age; laboratory parameters including alanine aminotransferase (ALT), hemoglobin, lactate dehydrogenase (LDH) and alkaline phosphatase (ALP); metastatic characteristics including number, size, response to chemotherapy and existence of extrahepatic metastasis; treatment criteria including number of chemotherapy cycles, radiotherapy of the primary tumor and local therapy of metastatic lesions were analyzed.

### Statistical analysis

OS was estimated by the Kaplan-Meier method. Statistical significance between survival curves was analyzed using the log-rank test. Multivariate analyses were performed using the Cox proportional hazards model to test for independent significance by backward elimination of insignificant explanatory variables. Covariates included patients’ characteristics, laboratory parameters, metastatic features and treatment criteria. The response rates were compared by chi-square test (χ^2^). A two-tailed *P*-value <0.05 was considered statistically significant.

## Results

### Patients’ clinicopathological characteristics

Out of the 91 patients who were initially referred to our center, six were excluded due to missing clinical or follow-up data. The clinicopathological data of the remaining 85 patients are presented in Table 1.

**Table 1:** Clinical characteristics

**Characteristics**	**N (%)**
Karnosky performance score (KPS)	
>70	74 (87.1)
≤70	11 (12.9)
Gender	
Male	78 (91.8)
Female	7 (8.2)
Age	
Median age	50 (28-75)
Alanine amino transferase ( ALT)(IU/I)	
>40	26 (30.6)
≤40	59 (69.4)
Hemoglobin (HB)(g/L)	
≥120	74 (87.1)
<120	11 (12.9)
Lactate dehydrogenase (LDH) (IU/l)	
>245	54 (63.5)
≤245	31 (36.5)
Alkaline phosphatase (ALP) (IU/l)	
>110	28 (32.9)
≤110	57 (67.1)
T stage (2002AJCC)	
T1-2	35 (41.1)
T3-4	50 (58.8)
N stage (2002AJCC)	
N0-1	21 (24.8)
N2	40 (47.1)
N3	24 (28.2)
No. of metastatic lesion	
≤3	35 (41.2)
>3	50 (58.8)
Size of metastatic lesions (cm)	
≤3	53 (62.3)
>3	32 (37.7)
With extrahepatic metastases	
Yes	49 (57.6)
No	36 (42.4)

### Treatment regimens and response

The treatment modalities, including response to chemotherapy, are described in Table 2. Seven patients refused treatment. The treatment modalities of the remaining 78 patients were determined according to the experience of our center and the acceptance of the patients. All 78 were treated with platinum-based chemotherapy. The median number of cycles was 6 (range: 1-15). A total of 32 patients received radiotherapy following chemotherapy. Radiotherapy of the primary tumor was generally administrated to those patients who achieved disease control of the metastatic lesions after chemotherapy. It was also administered to reduce serious symptoms caused by the primary tumor that affected the quality of life. Radiotherapy included treatment to the primary tumor and to the superclavicular lymph nodes; 26 patients received a radiation dose ≥66 Gy and six patients received a dose <66 Gy. Local therapy to metastases, including radiofrequency ablation (RFA), interventional embolization and liver radiotherapy (54 Gy/27f), was administered in 18 patients.

**Table 2:** Treatment characteristics

**Characteristics**	**N (%)**
Receiving treatment	
Yes	78 (91.7)
No	7 (8.3)
Radiotherapy of primary tumor	
Yes	32 (37.6)
No	53 (62.4)
Cycles of chemotherapy	
0	7 (8.2)
1-5	36 (42.3)
≥6	42 (49.4)
Chemotherapy regimen	
PF	42 (53.8)
TPF	21 (26.9)
TP	15 (19.2)
Local therapy of metastatic lesions	
Yes	18 (21.1)
RFA	13
Embolization	4
Radiotherapy	1
No	67 (78.9)
Response to chemotherapy	
Yes	52 (70.3)
No	22 (29.7)
No data	11

PF: Cisplatin + 5-Fu; TPF: Cisplatin + 5-Fu + Paclitaxel; TP: Cisplatin + Paclitaxe;RFA: Radiofrequency ablation.

### Toxicity

None of the patients exhibited grade V toxicity (death) during chemotherapy. However, 54.5% developed grade III–IV leucopenia or neutropenia, 28.5% developed grade II–III mucositis and 19.5% exhibited grade II–III toxicity with vomiting and nausea.

### Response and overall survival

Four patients were omitted from further assessments as imaging evaluations had not been performed after the first cycle of chemotherapy. Of the remaining 74 patients, 4/74 (5.4%) achieved complete response (CR), 48/74 (64.8%) achieved partial response (PR), 12/74 (16.2%) had stable disease (SD) and 10/74 (13.5%) had progressive disease (PD). The overall response and disease control rates were 70.4% and 86.4%, respectively.

Seventy-eight patients had died by the final evaluation date (December 31, 2012). The main cause of death was progression of metastatic lesions, which occurred in 74/78 (94.9%) patients; 3/78 (3.8%) patients died of local failure and 1/78 (1.3%) died of cardiac disease. The median survival time for all the patients was 19.0 months (range: 4-124 months). The 1-year, 2-year and 3-year survival rates were 71.8%, 34.1% and 14.1%, respectively. Long-term disease-free survival (>36 months) was achieved by five patients; their clinical characteristics are summarized in Table 3.

**Table 3:** Characteristics of the five patients who achieved long-term disease-free survival

**Age (years)**	**Pre-treatment status (KPS)**	**Pre-treatment LDH (IU/I)**	**No. of liver lesion**	**Chemotherapy**	**Radiotherapy**	**Local therapy of liver lesion**	**Survival time (months)**
29y	90	160	One	PF × 9 cycles	2D-RT	RFA	124
					70 Gy/35f		
40y	80	251	Four	PF × 10 cycles	2D-RT	No	117
					70 Gy/35f		
52y	90	206	Two	TP × 6 cycles	2D-RT	RFA	94
					74 Gy/37f		
65y	90	157	One	TPF × 6 cycles	2D-RT	RFA	91
					70 Gy/35f		
55y	80	206	Five	PF × 6 cycles	IMRT	No	54
					68 Gy/30f		

2D-RT: Two-Dimensional radiotherapy; IMRT: Intensity Modulate Radiotherapy.

### Univariate analyses

The results of the univariate analyses are summarized in Table 4. The negative prognostic factors for OS were as follows: KPS ≤70 (*P* < 0.01); LDH >245 IU/l (*P* < 0.01); ALT >40 IU/l (*P* < 0.01); number of metastatic lesions >3 (*P* = 0.01); occurrence of extrahepatic metastasis (*P* = 0.01); number of chemotherapy cycles <6 (*P* = 0.01); no response to chemotherapy (*P* < 0.01); no radiotherapy of the primary tumor (*P* < 0.01); and no local therapy of metastatic lesions (*P*=0.05).

**Table 4:** Univariate analysis of variables correlated with overall survival

**Characteristic**	**3-year OS (%)**	**HR (95% CI)**	***P*-value**
KPS: ≤70/>70	0.0 (16.2)	5.76 (3.09-10.69)	<0.01^a^
Gender: male/female	11.5 (28.6)	0.92 (0.59-1.43)	0.71
Age: >50/≤50 years	12.5 (15.6)	1.13 (0.53-1.30)	0.34
ALT(IU/I): >40 /≤40	3.8 (18.6)	2.17 (1.32-3.57)	<0.01^a^
Hemoglobin (g/L): <120/≥120	9.1 (14.9)	1.71 (0.87-3.36)	0.11
LDH (IU/I): ≥245/<245	9.3 (22.6)	1.67 (1.17-2.43)	<0.01^a^
ALP (IU/I): ≥110/<110	6.9 (16.1)	1.45 (0.91-2.31)	0.11
Number of lesions: >3/≤3	6.0 (25.7)	1.90 (1.19-3.03)	0.01^a^
Size of lesions (cm): >3/≤3	6.3 (18.9)	1.22 (0.76-1.94)	0.40
Extrahepatic metastases: yes/no	2.8 (22.4)	1.83 (1.16-2.89)	0.01^a^
Radiotherapy of primary tumor: no/yes	5.7 (28.1)	2.961 (1.79-4.75)	<0.01^a^
Cycles of chemotherapy: (1-5) vs. ≥6	8.6 (18.6)	2.20 (1.38-3.52)	0.01^a^
Response to chemotherapy: no/yes	0.0 (19.2)	4.03 (2.30-7.06)	<0.01^a^
Local therapy of lesions: no/yes	10.4 (27.8)	1.76 (1.02-3.04)	0.05^a^

^a^statistically significant; HR: hazard ratio; CI: confidence interval.

The following factors were significantly associated with poor response to chemotherapy: KPS ≤70 (*P* = 0.03); LDH >245 IU/l (*P* < 0.01); number of metastatic lesions >3 (*P* = 0.02); occurrence of extrahepatic metastasis (*P* = 0.04); and number of chemotherapy cycles <6 (*P* = 0.01).

### Multivariate analyses

**Figure 1:**
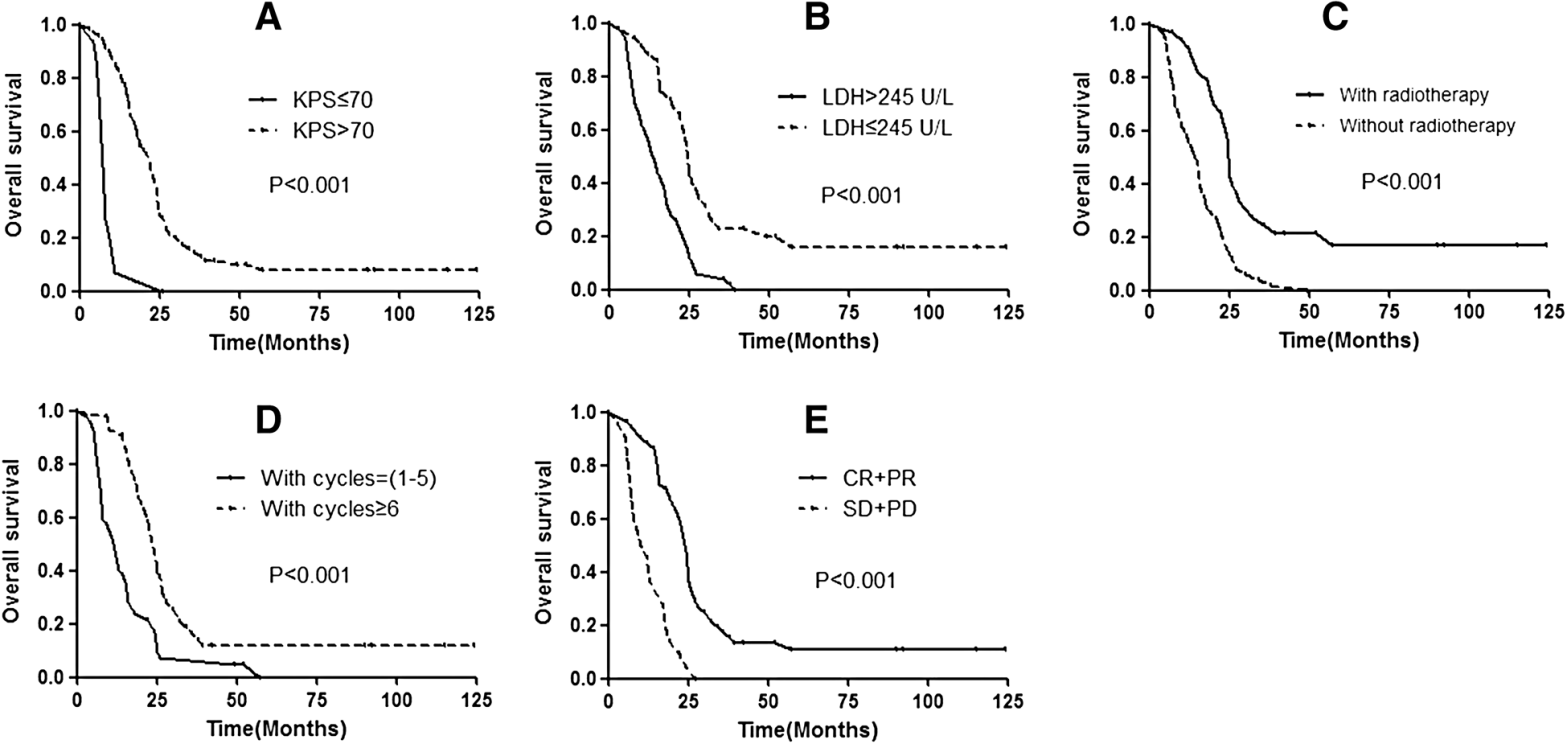
Overall survival rates according to KPS (A), LDH (B), radiotherapy of primary tumor (C), number of cycles of chemotherapy (D) and the response to chemotherapy (E).

The multivariate analysis results are summarized in Table 5. Significant factors for poor prognosis were KPS ≤70 (*P* = 0.04); LDH >245 IU/l (*P* = 0.01); no radiotherapy of the primary tumor (*P* < 0.01); number of chemotherapy cycles <6 (*P* < 0.01); and no response to chemotherapy (*P* < 0.01). These results are demonstrated in Figure 1.

**Table 5:** Multivariate analysis of variables correlated with overall survival

**Characteristic**	**HR (95% CI)**	***P*-value**
KPS: ≤70/>70	2.21 (1.1-4.90)	0.04
LDH (IU/I): ≥245/<245	2.09 (1.12-3.67)	0.01
Cycles of chemotherapy: (1-5) vs. ≥6	3.02 (1.65-5.56)	<0.01
Response to chemotherapy: no/yes	2.22 (1.29-3.49)	<0.01
Radiotherapy to primary tumor: no/yes	2.87 (1.61-5.10)	<0.01

Subgroup analysis showed that treatment with chemotherapy was also a positive prognostic factor for patients with KPS ≥ 80 (19.0% vs 10.7%, P = 0.02), however, no statistical significance was in patients with KPS ≤ 80 (P = 0.88). Significantly improved survival was also achieved by radiotherapy of the primary tumor in patients who achieved CR or PR after chemotherapy of metastatic lesions (30.8% vs. 3.8%, *P* < 0.01). In contrast, no significant difference was observed for patients with SD.

## Discussion

Liver metastasis is a common occurrence at initial diagnosis in patients with metastatic NPC. The survival time in these patients is poor, ranging between 13.0 and 17.0 months [8-10]. Similar to these reports, our analyses gave a median survival time of 19.0 months, and a 3-year OS rate of 14.1%, which is lower than those reported for lung or bone metastasis [11,12].

The benefits of systemic chemotherapy have been demonstrated in many studies [14-18]. Platinum-based combination treatment with two or three drugs achieves high response rates and is the most widely used regimen [17]. In this study, the patients who received chemotherapy had a median survival time of 20.0 months, compared to 9.0 months in patients who refused treatment.

In cases of metastatic NPC where chemotherapy is the only curative option, it is important that patients undergoing platinum-based combination therapy receive a sufficient number of cycles. A retrospective study involving 20 long-term disease-free survivors with metastatic NPC showed that approximately six cycles of chemotherapy were required [14]. Wang et al. also found that patients who received six cycles of chemotherapy survived longer than those receiving fewer cycles. Furthermore, they showed that the number of chemotherapy cycles was an independent prognostic factor in metastatic NPC [18]. Our results, based on univariate and multivariate analyses, were consistent with both these reports, however due to the retrospective nature of our study, further confirmation is required.

The application of radiotherapy to the primary tumor in NPC patients with synchronous liver metastasis remains controversial. It is generally considered unnecessary due to their short life expectancy and serious late complications. However, due to improvements in techniques, several studies have demonstrated that local control of primary tumors through radiotherapy can improve quality of life and contribute to prolonged survival. Yeh et al. showed that the 2-year OS rate in patients with metastatic NPC at diagnosis was 24.0% when they received radiotherapy alone, compared to 10% in those who received chemotherapy alone [6]. They also showed that local control of the primary tumor reduced necrosis, bleeding, nasal obstruction and severe headaches. Our study supported these findings by showing improved survival rates in patients who responded to chemotherapy of the metastatic lesions when radiotherapy of the primary tumor was administered. Taken together, these findings indicate that better local control may help reduce the tumor burden and lower the risks caused by progression or recurrence in NPC.

Local therapy of metastatic lesions may also prolong survival. Although local therapy is widely applied in patients with liver metastasis from colorectal cancer [19,20], its application in metastatic NPC remains limited. Pan et al. reported that the median survival of 11 patients with 1–3 metastatic lesions receiving treatment with RFA was 48.1 months, which was higher than those not receiving RFA [13]. Furthermore, procedure-related complications were infrequent. The treatment regimens for local therapy of liver metastases analyzed in this study included RFA, interventional embolization and liver radiotherapy. The median survival for all three modalities was 23.0 months and included three long-term disease-free survivors. RFA was found to be more effective than both interventional embolization and liver radiotherapy, giving a median survival time of 32.0 months compared to 9.0 months; however, this difference may be related to other factors between the patients. Patient with 1-3 metastatic lesions were found to benefit the most from RFA, with a median survival of 36.0 months compared to 21.0 months for those not undergoing RFA. Therefore, local therapy to metastatic liver lesions, particularly with RFA, should be considered in patients with NPC who have ≤3 lesions to further improve their survival when the primary tumor and metastatic diseases are stable.

Patients’ response to chemotherapy was found to be a significant prognostic factor. The overall response rate was 76.4% and was associated with performance status, LDH level, number of metastatic lesions, presence of extrahepatic metastasis and number of chemotherapy cycles, suggesting that these factors could be potential predictors of treatment response. Patients with LDH >245 IU/L, multiple organ metastasis and >3 liver lesions had lower rates of CR or PR to chemotherapy. In clinical practice, the response of metastatic lesions to chemotherapy is a key consideration in the choice of treatment; therefore, patients with CR or PR were recommended for radiotherapy of the primary tumor as this could significantly improve survival.

LDH is a glycolytic enzyme which reversibly catalyzes pyruvate to lactic acid under anaerobic conditions. Elevated levels of LDH are considered a negative prognostic factor in many solid tumors, including advanced NPC, and have been associated with large tumor burden, tumor extension and high risk of metastasis [21-25]. Serum LDH levels twice normal levels are rarely seen in loco-regional disease but are commonly observed in NPC patients with liver metastasis or multiple organ metastases. As such, they have been described as a negative prognostic factor [23]. Studies have found that patients with advanced NPC and elevated baseline LDH levels were more likely to develop liver metastasis following treatment [24], and elevated LDH levels were reported in over 55.0% of patients with metastatic NPC, with hazard ratios up to 1.8 [25]. In our study, >60.0% of patients had elevated levels of LDH; furthermore, these patients had significantly poorer 3-year OS rates compared to those without elevated LDH levels (9.3% compared to 22.6%). These pretreatment serum levels of LDH may be a potential prognostic indicator.

In conclusion, we identified five independent prognostic factors in NPC patients who initially presented with liver metastasis. These included pretreatment performance status, LDH level, radiotherapy of the primary tumor, the number of chemotherapy cycles and response to chemotherapy. Although survival rates in these patients remains poor, our findings suggest that selected patients may achieve improved survival by undergoing comprehensive treatment, including six or more systemic chemotherapy cycles and radiotherapy of the primary tumor. The application of local therapy to metastatic lesions, in particular by RFA, may also prolong survival.

## Competing interests

The authors declare that they have no competing interests.

## Authors’ contributions

HF and T YM contributed to conception and design of the study, and revised the manuscript; WFH and ZL contributed to analysis and interpretation of data, and drafted the manuscript and revised the manuscript. LS, GY and LTX participated in data acquisition and literature research. All authors read and approved the final manuscript.
